# Role of chance in familial aggregation of colorectal cancer

**DOI:** 10.1054/bjoc.2000.1717

**Published:** 2001-04

**Authors:** N Katballe, S M Bentzen, M Christensen, F P Wikman, T Ørntoft, S Laurberg

**Affiliations:** Surgical Research Unit 900, Department of Surgery L, Aarhus University Hospital, Aarhus Amtssygehus, Aarhus, Denmark; Biostatistics in Oncology, Gray Laboratory Cancer Research Trust, Mount Vernon Hospital, Northwood, Middx, HA6 2JR, UK; Dept. of Clinical Biochemistry, Aarhus University Hospital, Skejby Sygehus, Aarhus, Denmark

**Keywords:** HNPCC, hereditary non-polyposis colorectal cancer, Amsterdam criteria, incidence, coincidence

## Abstract

A prospective population-based study recorded family trees of 77 colorectal cancer patients younger than 50 years of age. Using mathematical modeling of population age-incidence data, we estimate that 1 (95% confidence limits 0 and 3) of these families is expected to meet the Amsterdam criteria I for HNPCC due to chance clustering of colorectal cancer. © 2001 Cancer Research Campaign http://www.bjcancer.com


*British Journal of Cancer* (2001) **84**(8), 1084–1086
© 2001 Cancer Research Campaign
doi: 10.1054/ bjoc.2001.1717, available online at http://www.idealibrary.com on IDEAL

### Role of chance in familial aggregation of colorectal cancer

**N Katballe[1], SM Bentzen[2], M Christensen[3], FP Wikman[3], T Ørntoft[3] and S Laurberg[1]**

[1]Surgical Research Unit 900, Department or Surgery L, Aarhus University Hospital, Aarhus Amtssygehus, Aarhus, Denmark; [2]Biostatistics in Oncology, Gray Laboratory Cancer Research Trust, Mount Vernon Hospital, Northwood, Middx HA6 2JR, UK; [3]Dept. of Clinical Biochemistry, Aarhus University Hospital, Skejby Sygehus, Aarhus, Denmark

**Summary** A prospective population-based study recorded family trees of 77 colorectal cancer patients younger than 50 years of age. Using mathematical modeling of population age-incidence data, we estimate that 1 (95% confidence limits 0 and 3) of these families is expected to meet the Amsterdam criteria I for HNPCC due to chance clustering of colorectal cancer. © 2001 Cancer Research Campaign http://www.bjcancer.com

**Keywords**: HNPCC; hereditary non-polyposis colorectal cancer; Amsterdam criteria; incidence; coincidence

In 1991 the International Collaborative Group on Hereditary Non Polyposis Colorectal Cancer (ICG-HNPCC) defined the Amsterdam I clinical criteria for identifying HNPCC families. The following 4 criteria must be fulfilled: 1) At least 3 family members in at least 2 generations should have histologically verified colorectal cancer; 2) One family member must be under the age of 50 at the time of diagnosis; 3) One family member must be first degree relative of the 2 others; 4) FAP must be excluded (Vasen et al, 1991). Based on a high incidence of CRC in the Western world, we hypothesized that coincidental clustering of CRC among relatives of young colorectal cancer patients might explain a considerable fraction of the families who fulfil the Amsterdam criteria I.

### PATIENTS AND METHODS

### Purpose

The aim of this study was to estimate the number of coincidental Amsterdam criteria I positive families in a sample of non-selected CRC patients younger than 50 years of age. We also wanted to estimate the expected proportion of coincidental Amsterdam criteria I positive families among young patients' families meeting the Amsterdam criteria I.

### Patients

The study was designed as a prospective population-based multicentre study. Consecutive Danish patients with primary adenocarcinoma of the colon and rectum diagnosed before the age of 50, with official address in a participating county, were invited to participate. Written informed consent was required to include a patient, and the study was approved by the local ethical committee. 4 Danish counties (population 1.4 million, 26% of the Danish population) were successively included in the study from November 1995 to October 1998. (County of Aarhus was included November 1995, County of Ribe – August 1996, County of Ringkoebing – March 1997 and County of Viborg was included August 1997.) All Danish CRC patients are diagnosed and treated in the public health care system, and lists of all CRC patients from the Institutes of Pathology covering the area were checked regularly to ensure that no CRC patients were missed.

### Family history

The surgeons filled in a questionnaire covering malignancy and age at time of diagnosis among family members, and the study group interviewed all patients about sex, current age or age at time of death for all first and second degree relatives. Hospital files, death certificates or information from the Danish Cancer Registry were requested on each family member with a possible HNPCC related cancer.

### Statistical methods

For each family the probability of meeting the Amsterdam Criteria I by chance was calculated using a specific FORTRAN program developed by the study group. The calculations were based on the age-and sex-specific incidence data from the Danish Cancer Registry for the year 1990 (Storm et al, 1994).

The Amsterdam criteria I, can be met through 7 different paths (Figure 1). Path 1 includes the patient, the parents and the grandparents. Path 2 includes the patient, the parents and the patient's children. It is clear that 5 additional paths exist, #3 to #7 in Figure 1.

The probability that a young colorectal cancer patient's family meets the Amsterdam criteria I by chance depends on the number of first and second degree relatives, their sex and current age or age at death and on the cumulative incidence rate of colorectal cancer in the background population.

If a patient is able to supply the above information for his/her parents and grandparents, the Amsterdam criteria I can be met in 4 different ways through path #1 (patient + patient's father + patient's father's father or father's mother or patient + patient's mother + patient's mother's father or mother). Information about any children generates new combinations through path #2. These

*Received 6 March 2000*
*Revised 9 November 2000*
*Accepted 23 January 2001*

*Correspondence to:* N Katballe

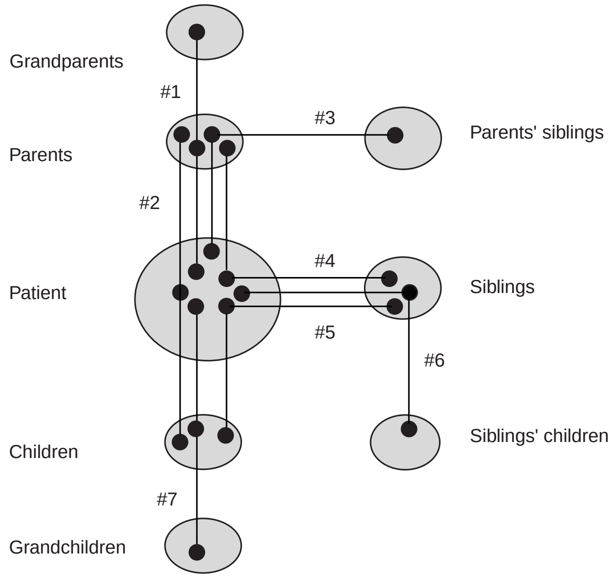

**Figure 1** The Amsterdam criteria I for hereditary non-polyposis colorectal cancer can be fulfilled through 7 paths (#1–#7).

principles continue through path #3–#7. The probabilities from the various paths are combined to yield the family's probability of meeting the Amsterdam criteria I.

### RESULTS

### Families meeting the Amsterdam criteria I

Among 1514 consecutive CRC patients, 87 (5.7%) were diagnosed before the age of 50. 77 patients (88.5%) were interviewed, and 4 families met the strict Amsterdam criteria I. Another 2 families did not meet the strict criteria due to lack of a verified diagnosis in a third family member. In one family, the third tumour was located at the splenic flexure detected on X-ray examination. Histology from metastasis showed adenocarcinoma. In another family the third family member had a big abdominal tumour and blood in the stool. Histology showed mucinous adenocarcinoma consistent with colorectal cancer. Hence for all practical purposes 6 families (6/77 = 7.8%) met the Amsterdam criteria I. By including the 2 family members described above, we observed 24 CRC cases in the 6 families, and all cases were histologically verified.

### Probability calculations

77 patients (77/87 = 88.5%) supplied information about current age or age at the time of death and sex among 1407 first and second degree relatives (an average of 18.3 members per family).

Every family's probability of meeting the Amsterdam criteria I by chance was calculated. The 77 families' risk of sporadically meeting the Amsterdam criteria I ranged from 0.0% to 7.1%, the average chance being 1.1%. 13 of the 77 families (17%) had a more than 2% risk of meeting the Amsterdam criteria I by coincidence.

In this sample of 77 families the estimated chance of sporadic fulfilment of the Amsterdam criteria I corresponds to an expectation of 0.83 family. The chances of finding at least 1, 2 or 3 Amsterdam I positive families follow a binomial distribution and these are 56.7%, 20.2% and 5.1% respectively. The probabilities of finding 4, 5 or 6 positive families are 1.0%, 0.2% and 0.02%, respectively. In this sample of 77 families, the median expected number of families meeting the Amsterdam criteria I by coincidence is 1 (95% confidence limits 0–3).

### DISCUSSION

In this study the chance of a family meeting the Amsterdam criteria I by coincidence, has been quantified for the first time on real families. 6 out of 77 families (7.8%) met the Amsterdam criteria I. We estimated a 0.02% probability of finding 6 or more families due to coincidental clustering in this material, so it is very unlikely that all 6 families are coincidental. Pathogenic mutations in the genes hMLH1 or hMSH2 are detected in approximately 50% of the families that meet the Amsterdam criteria I, and in 8–28% of suspected HNPCC families depending on which criteria of suspicion that are used (Buerstedde et al, 1995; Moslein et al, 1996; Viel et al, 1997; Wijnen et al, 1998; Park et al, 1999). Therefore we would not expect that all families would meet the criteria by coincidence.

Our calculations show, that when 77 young CRC patients are interviewed, there is a 56.7% chance of finding at least 1 family meeting the criteria. There is a 20.2% risk of finding 2 or more families by coincidence, and a 5.1% risk of finding 3 or more families that meet the criteria by coincidence. Hence, the most likely outcome is, that 1 out of our 6 families (17%), is the result of coincidental aggregation of CRC among family members.

The 6 families who met the Amsterdam criteria I were screened for hMLH1/hMSH2 mutations by SSCP, HD and PTT (data not shown). We have previously shown that the combination of using SSCP and HD are highly sensitive for the detection of hMLH1/hMSH2 mutations (Wikman et al, 2000). We detected mutations in 2 out of the 6 families, leaving us with 4 unexplained families. Therefore, the risk of meeting the Amsterdam criteria I by chance in mutation negative families is more likely 1 in 4 (25%) than the 1 in 6 (17%) risk that we have demonstrated on the Amsterdam criteria I positive families where mutational status is not taken into account.

The calculations are based on the assumption of a low proportion of hereditary CRC cases among the total number of CRC cases in Denmark and that environmental exposures and dietary habits are similar in Danish families.

We recommended all 6 families to follow the surveillance programme proposed by ICG-HNPCC. It is impossible to distinguish between HNPCC families and families who meet the criteria by coincidence, unless a pathogenic mutation has been detected.

Our results show that the majority of families of young CRC patients, who meet the Amsterdam criteria I are likely to be due to a genetic predisposition, but a considerable fraction of the families can be explained by chance aggregation of CRC in family members.

### ACKNOWLEDGEMENTS

We gratefully acknowledge the efforts of our colleagues at the participating surgical departments and institutes of pathology for filling in questionnaires, collecting tissue/blood samples and filling in forms to describe the cancers. In particular we want to thank Dr Peter Rasmussen, Dr Niels Krarup, Dr Ole Brandsborg, Dr Peter Saksø, Dr Knud Rasmussen, Dr Claus Nepper Holm, Dr NO Jacobsen, Dr Flemming Melsen, Dr Helle Dybdahl, Dr Niels

*© 2001 Cancer Research Campaign*

Ryegaard Rasmussen, Dr Anders Larsen, Dr Inger Marie Reintoft, Dr Mogens Rørbæk Madsen, Dr Erik Skoubo Kristensen, Dr Ivan Nøbæk Sørensen, Dr Asger Bisgaard Petersen, Dr Ebbe Fuglsang, Dr Max Vetner, Dr Jørn Nielsen, Dr Lausten-Thomsen, Dr Thor Knudsen, Dr Einar Holgersen and Dr Vibeke Thue Nielsen.

We are also very grateful to Dr Steffen Bülow for his contribution to this paper and to Dr Lone E Munk Sunde for her support in the genetic counselling of the families.

Financial support has been received from 'Karen Elise Jensen Foundation', Denmark, Institute of Experimental Clinical Research, Aarhus University, Denmark, 'The Danish Cancer Society', 'The Boel Foundation', Denmark, 'Dansk Kraeftforsknings Foundation', Denmark, 'Surgical Research Foundation' – Randers Hospital, Denmark, 'Snedkermester Sophus Jacobsen og hustru Astrid Jacobzsen Foundation', Denmark, 'Health Insurance Fond', Denmark, 'Fritz, Georg and Marie Cecilie Gluds legat', Denmark and 'Aarhus Amtssygehus research Foundation', Denmark.

### REFERENCES

Buerstedde JM, Alday P, Torhorst J, Weber W, Muller H and Scott R (1995) Detection of new mutations in six out of 10 Swiss HNPCC families by genomic sequencing of the hMSH2 and hMLH1 genes. *J Med Genet* **32**(11): 909–912

Moslein G, Tester DJ, Lindor NM, Honchel R, Cunningham JM, French AJ, Halling KC, Schwab M, Goretzki P and Thibodeau SN (1996) Microsatellite instability and mutation analysis of hMSH2 and hMLH1 in patients with sporadic, familial and hereditary colorectal cancer. *Hum Mol Genet* **5**(9): 1245–1252

Park JG, Vasen HF, Park KJ, Peltomaki P, Ponz dL, Rodriguez-Bigas MA, Lubinski J, Beck NE, Bisgaard ML, Miyaki M, et al. (1999) Suspected hereditary nonpolyposis colorectal cancer: International Collaborative Group on Hereditary Non-Polyposis Colorectal Cancer (ICG-HNPCC) criteria and results of genetic diagnosis. *Dis Colon Rectum* **42**(6): 710–715

Storm HH, Manders T, Friis S, et al. (1994) *Cancer incidence in Denmark 1990.* Danish Cancer Society

Vasen HF, Mecklin JP, Khan PM and Lynch HT (1991) The International Collaborative Group on Hereditary Non-Polyposis Colorectal Cancer (ICG HNPCC). *Dis Colon Rectum* **34**(5): 424–425

Viel A, Genuardi M, Capozzi E, Leonardi F, Bellacosa A, Paravatou-Petsotas M, Pomponi MG, Fornasarig M, Percesepe A, Roncucci L, et al. (1997) Characterization of MSH2 and MLH1 mutations in Italian families with hereditary nonpolyposis colorectal cancer. *Genes Chromosomes Cancer* **18**(1): 8–18

Wijnen JT, Vasen HF, Khan PM, Zwinderman AH, van der Klift H, Mulder A, Tops C, Moller P and Fodde R (1998) Clinical findings with implications for genetic testing in families with clustering of colorectal cancer. *N Engl J Med* **339**(8): 511–518

Wikman FP, Katballe N, Christensen M, Laurberg S and Orntoft TF (2000) Efficient mutation detection in mismatch repair genes using a combination of single-strand conformational polymorphism and heteroduplex analysis at a controlled temperature. *Genet Test* **4**(1): 15–21

 *© 2001 Cancer Research Campaign*
